# The Effect of Enzymatically Polymerised Polyphenols on CD4 Binding and Cytokine Production in Murine Splenocytes

**DOI:** 10.1371/journal.pone.0036025

**Published:** 2012-04-23

**Authors:** Daisuke Yamanaka, Yumi Tamiya, Masuro Motoi, Ken-ichi Ishibashi, Noriko N. Miura, Yoshiyuki Adachi, Naohito Ohno

**Affiliations:** Laboratory for Immunopharmacology of Microbial Products, School of Pharmacy, Tokyo University of Pharmacy and Life Sciences, Hachioji, Tokyo, Japan; Charité, Campus Benjamin Franklin, Germany

## Abstract

High-molecular weight polymerised polyphenols have been shown to exhibit anti-influenza virus, anti-HIV, and anti-cancer activities. The purpose of this study was to evaluate the immunomodulating activities of enzymatically polymerised polyphenols, and to clarify the underlying mechanisms of their effects. The cytokine-inducing activity of the enzymatically polymerised polyphenols derived from caffeic acid (CA), ferulic acid (FA), and *p*-coumaric acid (CoA) was investigated using murine splenocytes. Polymerised polyphenols, but not non-polymerised polyphenols, induced cytokine synthesis in murine splenocytes. Polymerised polyphenols induced several cytokines in murine splenocytes, with interferon-γ (IFN-γ) and granulocyte-macrophage colony-stimulating factor (GM-CSF) being the most prominent. The underlying mechanisms of the effects of the polymerised polyphenols were then studied using neutralising antibodies and fluorescent-activated cell sorting (FACS) analysis. Our results show that polymerised polyphenols increased IFN-γ and GM-CSF production in splenocytes. In addition, the anti-CD4 neutralised monoclonal antibody (mAb) inhibited polymerised polyphenol-induced IFN-γ and GM-CSF secretion. Moreover, polymerised polyphenols bound directly to a recombinant CD4 protein, and FACS analysis confirmed that interaction occurs between polymerised polyphenols and CD4 molecules expressed on the cell surface. In this study, we clearly demonstrated that enzymatic polymerisation confers immunoactivating potential to phenylpropanoic acids, and CD4 plays a key role in their cytokine-inducing activity.

## Introduction

Recently, various biological and pharmacological functions of polyphenols have been studied. For instance, epigallocatechin gallate (EGCG) is a well-known functional phenolic compound from tea leaves. Green tea polyphenols show various beneficial functions such as anti-obesity [1], anti-HIV [2], [3], antioxidative [4], anti-cancer [5], anti-mutagenic [6], and hypocholesterolaemic activities [7]. In addition, oolong tea polymerised polyphenols (OTPP), which are polymers of catechins, have been reported to suppress postprandial hypertriglyceridaemia [8]. Lignin, a naturally occurring high-molecular weight phenolic compound, has also been shown to have anti-tumour, anti-influenza virus, anti-HIV, and anti-herpes simplex virus activities [9]–[11]. Phenolic compounds derived from functional foods such as green tea leaf [12], grape seed [13], pomegranate fruit [14], and *Rhus verniciflua* [15] have been reported to inhibit immune systems through suppression of mitogen-activated protein kinases (MAPKs) and nuclear factor-κB (NF-κB) activation. Generally, low-molecular weight phenolic compounds, including EGCG and hydroxycinnamic acid derivatives, have anti-inflammatory effects [16]–[18]. However, the mechanisms of action of high-molecular polyphenols on immunomodulating functions in murine leukocytes have not been characterised in detail.

Numerous plants possess various polyphenols, and we regularly ingest polyphenol compounds as foods, which subsequently influence our health. Some polyphenols contained in functional foods and supplements are now used in alternative medicines. However, many foods contain not only phenolic compounds but also polyphenol-related enzymes such as polyphenol-oxidase and peroxidase. For instance, the edible mushroom, *Agaricus brasiliensis* (*Agaricus blazei*) [19] possesses potent polyphenol-oxidase and peroxidase activities in the fruiting body [20]; and its extract gradually changes to brown colour, because of their enzymatic action. These facts led us to hypothesise that enzymatically polymerised polyphenols could contribute, at least in part, to the various beneficial effects of *A. brasiliensis*, such as its anti-tumour activity and various immunoenhancing properties [21]–[24].

Phenolic compounds are easily converted to high-molecular weight polyphenols by various enzymes. Therefore, to facilitate the use of functional foods as alternative medicines, we decided to investigate whether high-molecular phenolic compounds exert immunomodulatory activities and their possible mechanisms. In the present study, we prepared polymerised polyphenols using horseradish peroxidase (HRP) and hydrogen peroxide as an enzymatic source, and investigated the effect of enzymatically polymerised representative phenylpropanoic acids such as CA, FA, and CoA on immunomodulating activity.

## Results

### Physicochemical characterisation of polymerised polyphenols

The polymerised polyphenols were synthesised from CA, *trans*-FA, and *trans*-CoA by the bulk method. The yield, protein contamination based on the nitrogen content, and endotoxin contamination in the polymerised caffeic acid (pCA), *trans*-ferulic acid (pFA), and *trans*-*p*-coumaric acid (pCoA) are summarised in Table 1. Both endotoxin and protein (HRP from the enzymatic source) contamination in polymerised polyphenols were either very low or not detectable.

**Table 1:** Yield, elemental analysis, and endotoxin content of samples.

		Elemental analysis (%)			Endotoxin
	Yield (%)	Carbon	Hydrogen	Nitrogen	(pg/mg)
CA	-	59.91	4.59	0.01	-
FA	-	61.80	5.20	0.01	-
CoA	-	65.91	4.91	0.01	-
pCA	40.0	46.45	4.15	0.02	231.5
pFA	35.2	53.75	5.01	0.00	93.7
pCoA	40.0	57.62	4.67	0.00	n.d.

-; not done.

n.d.; not detected.

Yields based on precursor weights.

### Effect of polyphenols on cytokine synthesis *in vitro*

**Figure 1:**
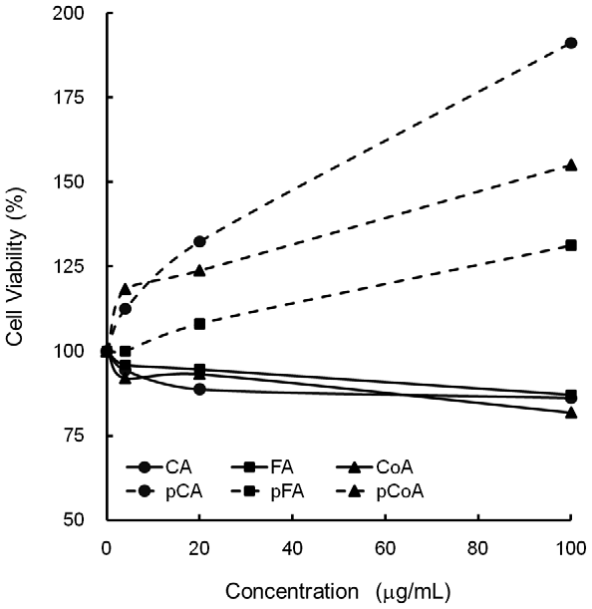
Effect of various polyphenols on cell viability in murine splenocytes. C57BL/6 splenocytes were stimulated with various polyphenols (0–100 µg/mL). After 48 h, relative living cell numbers were assessed by the MTT method. The vertical axis represents the percentage living cell numbers of the splenocytes, and was obtained on the basis of the ratio to the control cells. The values represent the mean ± standard deviation, n = 3.

**Figure 2:**
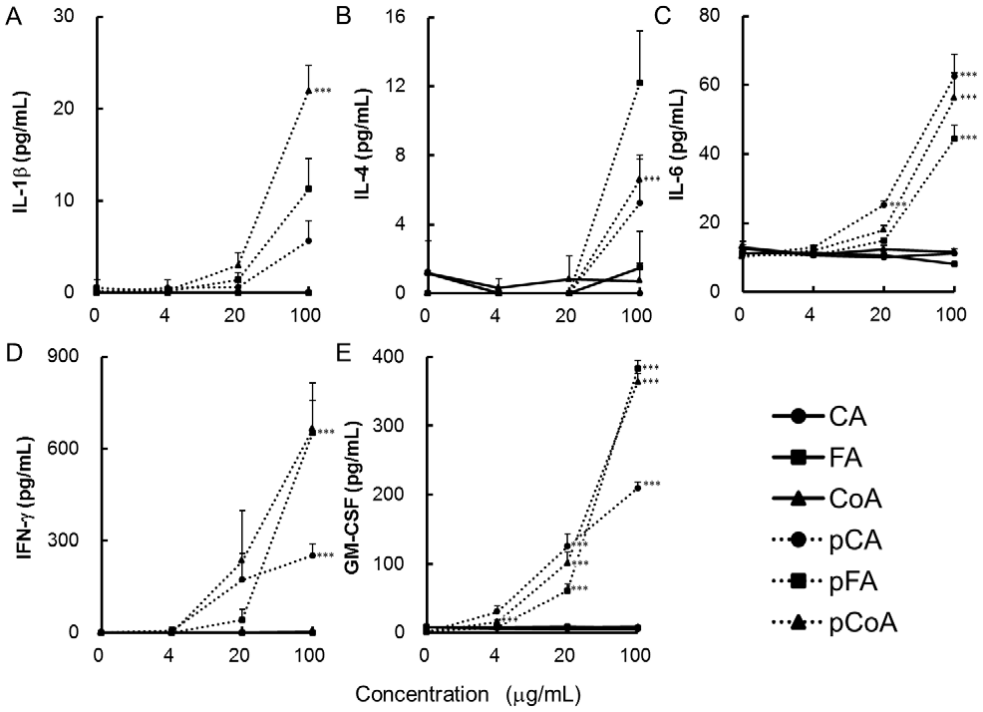
Effect of polymerised polyphenols on cytokine production in murine splenocytes. C57BL/6 splenocytes were stimulated with various polyphenols (0–100 µg/mL). After 48 h, supernatants were collected and concentrations of cytokines; (A) IL-1β, (B) IL-4, (C) IL-6, (D) IFN-γ, and (E) GM-CSF were measured by ELISA. The values represent the mean ± standard deviation, n = 3. Significant difference from untreated splenocytes: ***p<0.001.

To confirm the immunomodulatory activity of polymerised polyphenols, *in vitro* cell culture with C57BL/6 mouse splenocytes in the presence of various polyphenols was performed. We first examined the cytotoxicity of our polymerised polyphenol preparations on murine splenocytes using the MTT assay. As shown in Figure 1, polymerised polyphenols did not induce any cytotoxic effects up to a concentration of 100 µg/mL. Comparatively, polymerised polyphenols, but not monomers, induced the proliferation of splenocytes in a dose-dependent manner, as reported previously [25]. Next, we examined whether the polymerised polyphenols can induce cytokine production from murine splenocytes. Our results show that only the polymerised polyphenols, not the non-polymerised polyphenols, induced various cytokine productions from splenocytes. In particular, IFN-γ and GM-CSF production was strongly induced by the polymerised polyphenols in a dose-dependent manner (Figure 2). Taken together, these results clearly demonstrate that the polymerised polyphenols exhibit immunoenhancing activity against murine splenocytes, and that polymerisation is required for these activities.

### Involvement of T cells in cytokine production from splenocytes

**Figure 3:**
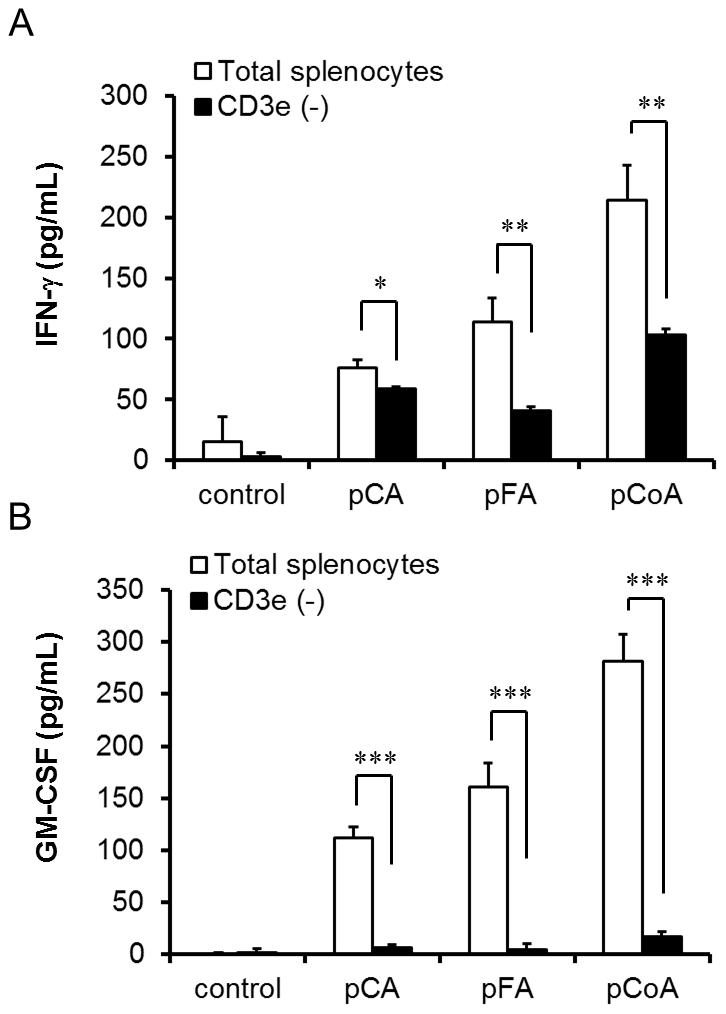
Contribution of T cell population to the polymerised polyphenol-induced cytokine production from murine splenocytes. Total splenocytes and CD3e^+^ cell-eliminated splenocytes were stimulated with various polymerised polyphenols (100 µg/mL). After 48 h, the supernatants were collected, and the concentrations of (A) IFN-γ and (B) GM-CSF were measured by ELISA. The values represent the mean ± standard deviation, n = 3. Significant difference from untreated splenocytes: *p<0.05; **p<0.01; ***p<0.001.

We then focused on the production of IFN-γ and GM-CSF as indicators of the bioactivity of polymerised polyphenols. In case of mitogenic activity of lignin, previous reports have indicated that T lymphocytes are the primary target for the DNA synthesis activity of lignin [25]. To investigate the underlying mechanisms of the effects of polymerised polyphenols on IFN-γ and GM-CSF production from murine splenocytes, we examined the effect of polymerised polyphenols on CD3e^+^ T cell-deficient splenocytes. Figure 3 shows that IFN-γ and GM-CSF production induced by polymerised polyphenols strongly decreased in the absence of these T cells, suggesting that the T cell population is crucial for IFN-γ and GM-CSF production induced by polymerised polyphenols.

### Cytokine synthesis induced by polymerised polyphenols is regulated by CD4

**Figure 4:**
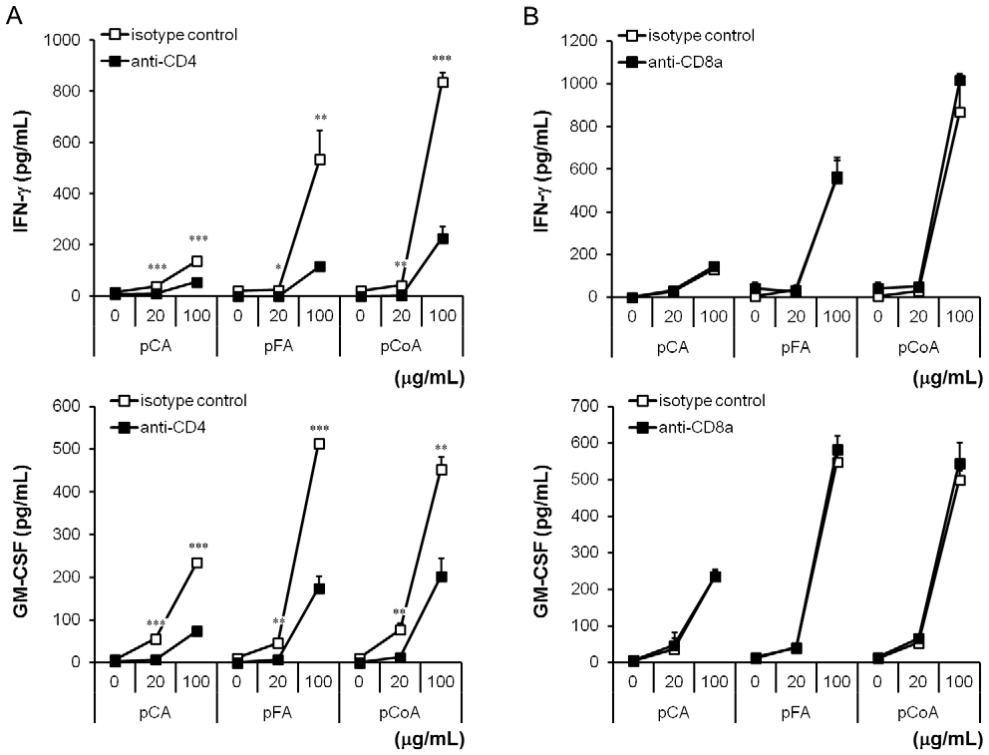
Involvement of CD4 in cytokine production induced by polymerised polyphenols from splenocytes. Splenocytes were pre-incubated with 1 µg/mL of (A) anti-CD4 mAb or (B) anti-CD8a mAb, and each control isotype (rat IgG2b and rat IgG2a; respectively) for 1 h, and then exposed to various polymerised polyphenols (0–100 µg/mL). After 48 h of incubation, the supernatant was collected, and the concentrations of IFN-γ and GM-CSF were determined by ELISA. The values shown represent the mean ± standard deviation, n = 3. Significant difference from isotype control: *p<0.05; **p<0.01; ***p<0.001.

On the basis of the above-described results, we focused our attention on the representative T cell surface receptors, namely, CD4 and CD8. We next examined the effects of neutralising mAb against CD4 and CD8 with respect to IFN-γ and GM-CSF production in C57BL/6-derived splenocytes. Our results show IFN-γ and GM-CSF production induced by pCA, pFA, and pCoA was significantly suppressed by the pre-treatment of anti-CD4 mAb, but not anti-CD8a mAb, indicating that IFN-γ and GM-CSF production in murine splenocytes by polymerised polyphenols might be modulated by CD4 (Figure 4).

**Figure 5:**
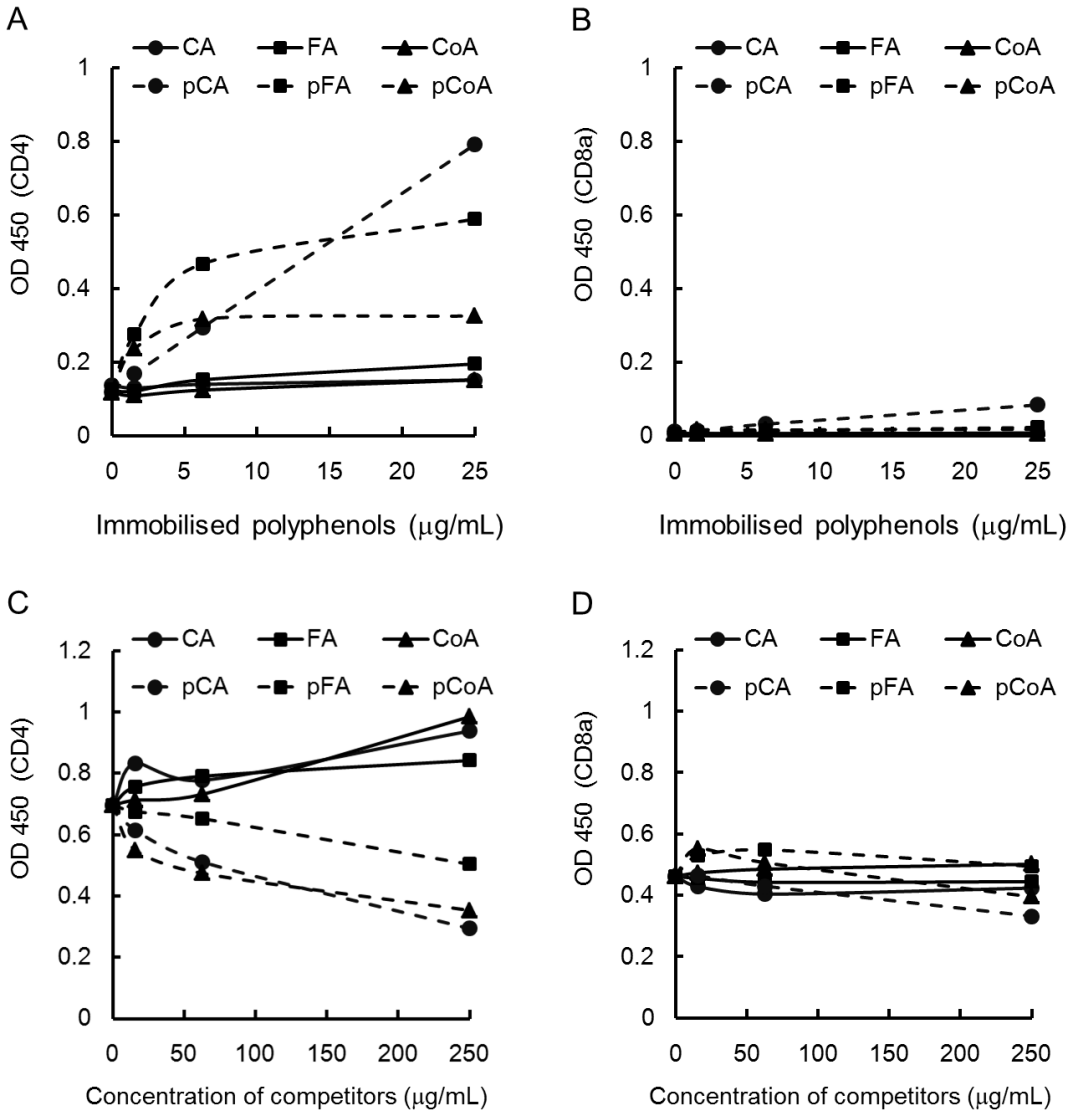
Binding capacity of the polymerised polyphenols to CD4 molecules. Each well of the ELISA plate was coated with various polyphenols (0–25 µg/mL) and blocked. Polyphenols bound to the wells were incubated with His-tagged (A) CD4 or (B) CD8a protein, washed, and probed with peroxidase-conjugated anti-6-His antibody. The binding affinity of the CD4 or CD8a molecule for the polyphenols was assessed using a colorimetric assay with the peroxidase substrate TMB and phosphate. The absorbance was measured at 450 nm. Reverse ELISA experiment was performed by coating the ELISA plate with (C) His-tagged CD4 or (D) CD8a protein and blocking. The plate was incubated with various polyphenols diluted to achieve concentrations of 0–250 µg/mL, washed, and further treated with anti-CD4 or CD8a mAb. The plate was probed with peroxidase-conjugated anti-Rat IgG antibody; the binding of anti-CD4 or CD8a mAb to solid-phase CD4 or CD8a protein was monitored using TMB, and the absorbance was measured as mentioned above.

**Figure 6:**
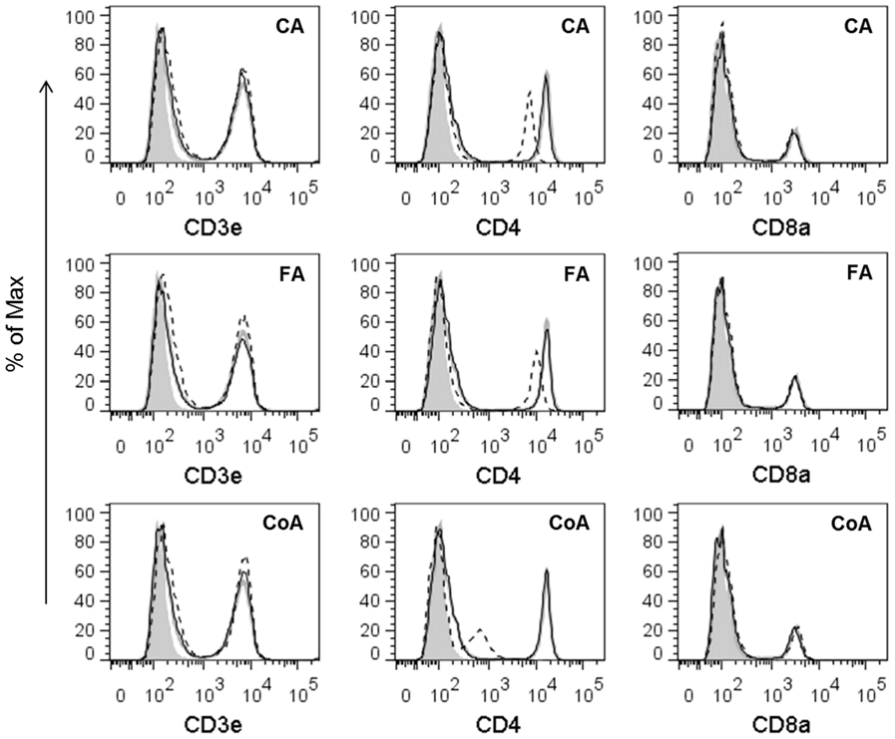
The competitive binding of polyphenols and anti-CD4 antibody to cell-surface CD4. Fresh splenocytes were pre-incubated with various polyphenols. After blocking the Fc receptors, splenocytes were incubated with anti-CD3e-PE, anti-CD4-APC, anti-CD8a-FITC, or isotype-matched control mAbs. The interaction between cell-surface receptors and monoclonal antibodies was examined using FACS. Grey shading represents the control cells with control antibody; grey lines represent the control cells with functional antibody; black continuous lines represent the monomer phenylpropanoic acids-treated cells with functional antibody; and black dashed lines represent the polymerised polyphenols-treated cells with functional antibody. The data shown are representative of 3 independent experiments.

We also investigated the capacity of polymerised polyphenols to bind to the murine CD4 by an ELISA-like assay and FACS. As shown in Figure 5A, immobilised polymerised polyphenols significantly increased the absorbance when reacted with CD4; however, non-polymerised polyphenols showed no effect. On the other hand, immobilised polymerised polyphenols did not bind to soluble CD8a protein (Figure 5B). To confirm that the above-mentioned action of polymerised polyphenols was not due to the differences in their abilities to bind the ELISA plate, a reverse experiment in which a competitive ELISA assay was performed using solid-phase CD4 and anti-mouse CD4 mAb. Anti-CD4 mAb binding to solid-phase CD4 protein was inhibited by polymerised polyphenols in a dose-dependent manner. Comparatively, monomers did not interfere with the binding of anti-CD4 mAb to immobilised CD4 (Figure 5C). In addition, polyphenols did not interfere with the binding of anti-CD8a mAb to immobilised CD8a (Figure 5D). Furthermore, the binding capacity of polymerised polyphenols to cell surface CD4 was examined. Polymerised polyphenols, but not monomers, strongly inhibited the binding of anti-mouse CD4 mAb to the CD4 expressed on the cell surface of splenocytes (Figure 6). Conversely, polymerised polyphenols did not inhibit the binding of anti-mouse CD3e and anti-mouse CD8a to the CD3e and CD8a expressed on the cell surface. Taken together, our results strongly suggest that the polymerised polyphenols bind specifically to CD4 molecules and that CD4 plays a key role in induction of IFN-γ and GM-CSF expression.

## Discussion

In the present study, we clearly demonstrate that polymers, but not monomers, of phenylpropanoids induce cytokine production from murine splenocytes. Furthermore, polymerised polyphenols directly bind CD4, and inhibition of CD4 function significantly suppresses IFN-γ and GM-CSF production in murine splenocytes; indicating that the immunomodulating effects of polymerised polyphenols are regulated, at least in part, by the CD4 molecule.

Several reports have recently shown that hydroxycinnamic acid derivatives exert anti-inflammation activity through the suppression of NF-κB [17], [26], [27]. However, polyphenol oxidase and peroxidase often interfere with phenolic compounds contained in foods, resulting in polymerisation. Therefore, we consume high-molecular weight polymerised polyphenols in our daily diet. Consequently, revealing the immunomodulating activities and their underlying mechanisms induced by polymerised polyphenols is crucial. Figures 1 and 2 show that polymerised phenylpropanoic acids, but not monomers, induce cell proliferation and cytokine production, especially IFN-γ and GM-CSF, in murine splenocytes; implying that enzymatic polymerisation can confer immunoactivating properties to phenylpropanoic acids. Our results provide new insights into the functions of polyphenols.

Both IFN-γ and GM-CSF are known to play central roles in innate immunity. IFN-γ is closely related to natural killer (NK) cell-associated anti-tumour activity [28]–[30]. On the other hand, GM-CSF is essential for anti-microbial activities against viruses, bacteria, and fungi [31], [32]. As for functional foods, IFN-γ and GM-CSF are important factors in the treatment of cancer and prevention of infectious diseases. Many functional foods, which are used in complementary and alternative medicines for cancer therapy, have the ability to induce the activation of the host immune system. In Japan, some mushrooms are widely used as a health food, thereby expecting pharmacological activity [24], [33], [34]. The fruiting body of an edible Basidiomycetes mushroom, *A. brasiliensis*, rich in β-glucan induces NK cell activation through an IFN-γ-dependent pathway [23], [35]. Moreover, research has revealed that GM-CSF is required for the immunoenhancing activities induced by fungal-derived β-glucans [36]–[38], and that this mushroom has polyphenol-related enzymes such as polyphenol oxidase, and peroxidase [39], [40]. These enzymes could catalyse the polymerisation of phenolic compounds and activate the innate immune system through IFN-γ and GM-CSF production. Therefore, it is possible that the strong induction of immune activation in these foods requires the combination of high-molecular weight phenolic compounds and β-glucan. It will be important to investigate this relationship as well as the other possible relationships in the future.

In mice, the CD4 molecule is well known as a primary receptor expressed not only on the T-helper cells [41], [42] but also bone marrow myeloid [43] and splenic dendritic cells [44]. Moreover, it has been reported that CD4 in human monocytes acts as a signalling molecule for the induction of calcium flux and for the activation of protein kinase C [45]. We demonstrated here that polymerised phenylpropanoic acids induced IFN-γ and GM-CSF production from murine splenocytes, and that the T cell population and the CD4 molecule are important for the induction of cytokine activity (Figures 3, 4, 5, 6). Several polyphenols have been reported to bind to CD4 molecules. For instance, EGCG has demonstrated anti-HIV activity through binding to CD4 and interfering with gp120 binding [46]. In addition, part of the anti-HIV activity of lignins was shown by inhibition of CD4, which is involved in the entry of HIV into the cells [10], [47]. Our results here show that polymerised phenylpropanoic acids bind directly to the CD4 molecule and the specificity of this binding was suggested by the result that polymerised polyphenols did not bind to the CD8a and CD3e molecule (Figures 5 and 6). High-molecular-weight polyphenols could induce the polymerisation of CD4 molecules on a limited area of cell surface and lead to subsequent cell–cell interaction, resulting in the activation of the immune system. In addition, the immune activation induced by polymerised polyphenols might be mediated by T cell-dependent and T cell-independent mechanisms, because the IFN-γ and GM-CSF production induced by polymerised polyphenols was observed even in the absence of T cells from splenocytes. Thus, further investigation is needed to reveal the mechanisms of polymerised polyphenols and IFN-γ and GM-CSF, as well as to identify the cells secreting cytokines. In addition, it is important to determine whether enzymatically polymerised polyphenols are responsible for cytokine induction, and have potential for use in immunological applications.

In conclusion, this study indicates that the polymerised polyphenols synthesised by enzymes, but not monomers, strongly induce cytokine production from murine splenocytes. These results are important, and therefore, further work is required to elucidate the intricacies of these immunomodulating effects exhibited by polymerised polyphenols. Our findings contribute to understanding the mechanisms by which foods induce immunomodulating activity.

## Materials and Methods

### Ethics Statement

All animal experiments followed the guidelines for laboratory animal experiments provided by the Tokyo University of Pharmacy and Life Sciences, and each experimental protocol was approved by the Committee for Laboratory Animal Experiments at Tokyo University of Pharmacy and Life Sciences (P11–49).

### Animals and materials

Male C57/BL6 mice were purchased from Japan SLC (Shizuoka, Japan). The mice were principally housed in a specific pathogen-free environment, and were used at 7–9 weeks of age. Type II HRP was purchased from Sigma (MO, USA). We purchased 3,4-dihydroxycinnamic acid (CA), *trans*-4-hydroxy-3-methoxycinnamic acid (*trans*-FA), and *trans*-4-hydroxycinnamic acid (*trans*-CoA) from Tokyo Chemical Industry Co., Ltd, Tokyo, Japan, and 3-(4,5-dimethyl-2-thiazolyl)-2,5-diphenyl-2*H*-tetrazolium bromide (MTT), from Dojindo (Kumamoto, Japan). Anti-mouse CD4 (GK1.5) (rat IgG2b), anti-mouse CD8a (53-6.7) (rat IgG2a), rat IgG2a isotype control, anti-mouse CD16/CD32 (2.4G2) (Fc Block), FITC-conjugated anti-mouse CD8a (53-6.7) (rat IgG2a), FITC-conjugated rat IgG2a isotype control, PE-conjugated anti-mouse CD3e (145-2C11) (Armenian hamster IgG1), PE-conjugated hamster IgG1 isotype control, APC-conjugated anti-mouse CD4 (RM4–5) (rat IgG2a), and APC-conjugated rat IgG2a isotype control were purchased from BD Pharmingen (San Diego, CA). Rat IgG2b isotype control was obtained from eBioscience (San Diego, CA).

### Preparation of polymerised polyphenols

We performed enzymatic synthesis of the lignin-like component by using HRP and 3 types of phenylpropanoic acid (CA, FA, and CoA) as enzyme and precursors, respectively. The basic synthesis method described by Sakagami *et al.* was used with slight modifications [48]. Briefly, 200 mg of precursor phenylpropanoic acid was neutralized with 1 N NaOH and diluted to 10 mL with phosphate-buffered saline (PBS) containing 1 mg of HRP. The H_2_O_2_ solution (30%) was diluted to 0.1% with PBS, and 1.5 mol eq H_2_O_2_ to the phenylpropanoid was added drop wise into a mixture of precursor and HRP solution for a period of 1 h with stirring at room temperature. This combined reaction mixture continued to be stirred for 2 h at room temperature, and then, the mixture was treated with heat for 20 min at 100°C to inactivate HRP. After centrifugation, the supernatant was collected and extensively dialysed (MWCO: 50000) against distilled water for 2 d, and then lyophilised. The amount of endotoxin was evaluated by a quantitative limulus amoebocyte lysate assay. The activation of factor C (limulus reactivity) by polymerised polyphenol was measured using a chromogenic method with an endotoxin-specific reagent (Endospecy ES-50M Set) and standard endotoxin (Standard Endotoxin CSE-L Set; from *Escherichia coli* O113:H10 strain) (Seikagaku Corp., Tokyo, Japan). Elemental analysis of each polymerised polyphenol was conducted at the Laboratory for Analytical Chemistry, Tokyo University of Pharmacy and Life Sciences. All samples were dissolved in dimethylsulphoxide (20 mg/mL), and further diluted with saline before use in cell culture.

### Cell preparation

The spleen was isolated from mice and teased apart in RPMI 1640 medium. After centrifugation, the single cell suspension was treated with ACK-lysing buffer (8.29 g/L NH_4_Cl, 1 g/L KHCO_2_, 37.2 mg/L EDTA/2Na) to lyse red blood cells. After centrifugation, cells were maintained in RPMI 1640 medium supplemented with 50 µg/mL gentamicin sulphate (Sigma) and 10% heat-inactivated fetal bovine serum (FBS; Equitech-Bio, Texas, USA). Cells were cultured in 48-well flat-bottomed plates at 2×10^6^ cells/well in 0.5 mL of culture medium for cytokine assay, or cultured in 96-well flat-bottomed plates at 4×10^5^ cells/well in 0.1 mL of culture medium for cell proliferation assay, and stimulated with various polyphenols (0–100 µg/mL). Splenocytes were cultured at 37°C for 48 h in a humidified atmosphere containing 5% CO_2_ and 95% air.

### Elimination of T cells

Freshly isolated splenocytes were incubated with anti-CD3e microbeads (Miltenyi Biotec, Germany) and an LD column (Miltenyi Biotec) was prepared. CD3e^+^ cells were eliminated by magnetic-activated cell sorting (MACS) (Miltenyi Biotec), according to the manufacturer's instructions. Flow cytometric analysis determined the total splenocytes contained >25% T cells, while the T cell-depleted population possessed <1%.

### Cytokine assay

The culture supernatants obtained after the cells were stimulated with various polyphenols for 48 h were used for the cytokine assay. The cytokine concentrations in the supernatants were determined using an OptEIA kit (BD Biosciences). The data were expressed as the mean ± standard deviation for the samples assayed in triplicate. At least 3 independent experiments were conducted.

### Cell proliferation assay

The cytotoxicity of the polyphenols on murine splenocytes was determined by a previously reported method with slight modifications [49]. After stimulation with various polyphenols (0–100 µg/mL) for 2 d at 37°C, the splenocytes were centrifuged and washed twice with fresh RPMI 1640 medium and grown in 0.5 mg/mL MTT (dissolved in PBS and filtered through a 0.2 mm membrane) at 37°C. Four hours later, the intracellular formazan crystals were dissolved in dimethylsulphoxide, and the absorption values were measured at 550 nm. The absorption values were expressed as the cell proliferation rate (%), according to the control group as 100%.

### Soluble CD4-binding assay

The ability of the polymerised polyphenols to directly bind to mouse CD4 was assessed by an ELISA-like assay. An ELISA plate (Greiner Bio-one, Germany) was coated with various polyphenols (0–25 µg/mL) in 0.1 M sodium carbonate buffer (pH 9.5), and incubated overnight at 4°C. The plate was washed with PBS containing 0.05% Tween 20 (PBST) and blocked with 1% BSA-PBST (BPBST) at room temperature for 1 h. After washing, the plate was incubated at 37°C for 1 h with His-tagged recombinant mouse CD4 or CD8a protein (Sino Biological Inc., Beijing, China) (2 µg/mL) in BPBST. The plate was then washed with PBST, and treated with peroxidase-conjugated anti-6-His antibody (R&D Systems, Minneapolis, MN) in BPBST. The binding of CD4 or CD8a to solid-phase polyphenols was monitored using the peroxidase substrate TMB (KPL Inc., MD, USA), and colour development was stopped with 1 M phosphoric acid; the optical density was measured at 450 nm.

To exclude the possibility that results were because of the different binding capacities of the various polyphenols to the ELISA plate, the plate was coated with recombinant mouse CD4 protein, and the binding ability of anti-mouse CD4 mAb to immobilised CD4 treated with various concentrations of polyphenols was tested by competitive ELISA assay. The ELISA plate was coated with His-tagged recombinant mouse CD4 or CD8a protein (0.5 µg/mL) dissolved in 0.1 M sodium carbonate buffer (pH 9.5), and incubated overnight at 4°C; the unbound antibody was removed by washing, and the plate was blocked with BPBST. The plate was incubated for 30 min with various polyphenols diluted with BPBST to achieve concentrations of 0–250 µg/mL. After washing, the plate was incubated at room temperature for 1 h with anti-mouse CD4 mAb (GK1.5) or anti-mouse CD8a mAb (53-6.7) (0.5 µg/mL) in BPBST. The plate was then washed with PBST, and treated with peroxidase-conjugated anti-Rat IgG (H+L) goat polyclonal antibody (Wako Pure Chemical Industries, Ltd, Osaka, Japan) in BPBST. The binding of mAb to CD4 or CD8a protein was monitored by a colorimetric assay using the peroxidase substrate TMB. The absorbance at 450 nm was measured using a microplate reader (MTP450; Corona Electric Co., Ibaraki, Japan).

### Flow cytometric analysis of cell surface CD4 and polyphenol interaction

The prepared single cell suspensions of splenocytes (4×10^6^ cells/mL) in RPMI 1640 medium containing 10% FBS were incubated with various polyphenols (100 µg/mL) at 37°C for 1 h. After incubation, the cells were washed once in staining buffer (1% FBS and 0.09% sodium azide in PBS) and resuspended in staining buffer (1×10^6^ cells/100 µL) containing Fc block (1 µg/10^6^ cells), and further incubated on ice for 20 min. For cell surface staining, anti-CD3e-PE, anti-CD4-APC, anti-CD8a-FITC, or isotype-matched control mAbs, were added; the cells were incubated in the dark on ice for 30 min. After incubation, cells were washed twice in staining buffer, and fixed in formalin solution (10% formaldehyde in PBS). Flow cytometry was done using a FACSCanto flow cytometer (BD Biosciences), and data were analysed by FACSDiva (BD Biosciences) and FlowJo (Tree Star Inc., USA) software. Dead cells were excluded according to their forward and side scatters.

### Statistical analysis

The significance of the differences between the means was assessed using the Student's t-tests.

## References

[1] Murase T, Nagasawa A, Suzuki J, Hase T, Tokimitsu I (2002). Beneficial effects of tea catechins on diet-induced obesity: stimulation of lipid catabolism in the liver.. Int J Obes Relat Metab Disord.

[2] Fassina G, Buffa A, Benelli R, Varnier OE, Noonan DM (2002). Polyphenolic antioxidant (−)-epigallocatechin-3-gallate from green tea as a candidate anti-HIV agent.. AIDS.

[3] Yamaguchi K, Honda M, Ikigai H, Hara Y, Shimamura T (2002). Inhibitory effects of (−)-epigallocatechin gallate on the life cycle of human immunodeficiency virus type 1 (HIV-1).. Antiviral Res.

[4] Benzie IF, Szeto YT (1999). Total antioxidant capacity of teas by the ferric reducing/antioxidant power assay.. J Agric Food Chem.

[5] Milligan SA, Burke P, Coleman DT, Bigelow RL, Steffan JJ (2009). The green tea polyphenol EGCG potentiates the antiproliferative activity of c-Met and epidermal growth factor receptor inhibitors in non-small cell lung cancer cells.. Clin Cancer Res.

[6] Yen GC, Chen HY (1995). Antioxidant activity of various tea extracts in relation to their antimutagenicity.. J Agric Food Chem.

[7] Ikeda I, Kobayashi M, Hamada T, Tsuda K, Goto H (2003). Heat-epimerized tea catechins rich in gallocatechin gallate and catechin gallate are more effective to inhibit cholesterol absorption than tea catechins rich in epigallocatechin gallate and epicatechin gallate.. J Agric Food Chem.

[8] Toyoda-Ono Y, Yoshimura M, Nakai M, Fukui Y, Asami S (2007). Suppression of postprandial hypertriglyceridemia in rats and mice by oolong tea polymerised polyphenols.. Biosci Biotechnol Biochem.

[9] Nagata K, Sakagami H, Harada H, Nonoyama M, Ishihama A (1990). Inhibition of influenza virus infection by pine cone antitumor substances.. Antiviral Res.

[10] Lai PK, Donovan J, Takayama H, Sakagami H, Tanaka A (1990). Modification of human immunodeficiency viral replication by pine cone extracts.. AIDS Res Hum Retroviruses.

[11] Fukuchi K, Sakagami H, Ikeda M, Kawazoe Y, Oh-Hara T (1989). Inhibition of *herpes simplex* virus infection by pine cone antitumor substances.. Anticancer Res.

[12] Hirao K, Yumoto H, Nakanishi T, Mukai K, Takahashi K (2010). Tea catechins reduce inflammatory reactions via mitogen-activated protein kinase pathways in toll-like receptor 2 ligand-stimulated dental pulp cells.. Life Sci.

[13] Gessner DK, Ringseis R, Siebers M, Keller J, Kloster J (2011). Inhibition of the pro-inflammatory NF-kappaB pathway by a grape seed and grape marc meal extract in intestinal epithelial cells.. J Anim Physiol Anim Nutr (Berl).

[14] Rasheed Z, Akhtar N, Anbazhagan AN, Ramamurthy S, Shukla M (2009). Polyphenol-rich pomegranate fruit extract (POMx) suppresses PMACI-induced expression of pro-inflammatory cytokines by inhibiting the activation of MAP Kinases and NF-kappaB in human KU812 cells.. J Inflamm (Lond).

[15] Jung CH, Kim JH, Hong MH, Seog HM, Oh SH (2007). Phenolic-rich fraction from *Rhus verniciflua* Stokes (RVS) suppress inflammatory response via NF-kappaB and JNK pathway in lipopolysaccharide-induced RAW 264.7 macrophages.. J Ethnopharmacol.

[16] Singh R, Akhtar N, Haqqi TM (2010). Green tea polyphenol epigallocatechin-3-gallate: inflammation and arthritis.. Life Sci.

[17] Nagasaka R, Chotimarkorn C, Shafiqul IM, Hori M, Ozaki H (2007). Anti-inflammatory effects of hydroxycinnamic acid derivatives.. Biochem Biophys Res Commun.

[18] Jung WK, Choi I, Lee DY, Yea SS, Choi YH (2008). Caffeic acid phenethyl ester protects mice from lethal endotoxin shock and inhibits lipopolysaccharide-induced cyclooxygenase-2 and inducible nitric oxide synthase expression in RAW 264.7 macrophages via the p38/ERK and NF-kappaB pathways.. Int J Biochem Cell Biol.

[19] Wasser SP, Didukh MY, de A Amazonas MLA, Nevo E, Stamets P (2002). Is a widely cultivated culinary-medicinal Royal Sun Agaricus (the Himematsutake mushroom) indeed *Agaricus blazei* Murrill?. Int J Med Mushr.

[20] Hashimoto S, Akanuma AM, Motoi M, Imai N, Rodrignes CA (2006). Effect of culture conditions on the chemical composition and biological activities of *Agaricus brasiliensis* S. Wasser et al. (Agaricomycetideae).. Int J Med Mushr.

[21] Ohno N, Akanuma AM, Miura NN, Adachi Y, Motoi M (2001). (1→3)-beta-d-glucan in the fruit bodies of *Agaricus blazei*.. Pharmaceutical and Pharmacological Letters.

[22] Ohno N, Furukawa M, Miura NN, Adachi Y, Motoi M (2001). Antitumor beta glucan from the cultured fruit body of *Agaricus blazei*.. Biol Pharm Bull.

[23] Liu Y, Fukuwatari Y, Okumura K, Takeda K, Ishibashi KI (2008). Immunomodulating activity of *Agaricus brasiliensis* KA21 in mice and in human volunteers.. Evid Based Complement Alternat Med.

[24] Yamanaka D, Motoi M, Ishibashi K, Miura NN, Adachi Y (2012). Effect of *Agaricus brasiliensis*-derived cold water extract on Toll-like receptor 2-dependent cytokine production in vitro.. Immunopharmacol Immunotoxicol.

[25] Kurakata Y, Sakagami H, Oh-Hara T, Kawazoe Y, Asano K (1990). Mitogenic activity of natural and synthetic lignins against cultured splenocytes.. In Vivo.

[26] Tucsek Z, Radnai B, Racz B, Debreceni B, Priber JK (2011). Suppressing LPS-induced early signal transduction in macrophages by a polyphenol degradation product: a critical role of MKP-1.. J Leukoc Biol.

[27] Moon MK, Lee YJ, Kim JS, Kang DG, Lee HS (2009). Effect of caffeic acid on tumor necrosis factor-alpha-induced vascular inflammation in human umbilical vein endothelial cells.. Biol Pharm Bull.

[28] Gidlund M, Orn A, Wigzell H, Senik A, Gresser I (1978). Enhanced NK cell activity in mice injected with interferon and interferon inducers.. Nature.

[29] Djeu JY, Stocks N, Zoon K, Stanton GJ, Timonen T (1982). Positive self regulation of cytotoxicity in human natural killer cells by production of interferon upon exposure to influenza and herpes viruses.. J Exp Med.

[30] Weigent DA, Stanton GJ, Johnson HM (1983). Interleukin 2 enhances natural killer cell activity through induction of gamma interferon.. Infect Immun.

[31] Freund M, Kleine HD (1992). The role of GM-CSF in infection.. Infection.

[32] Weiss M, Belohradsky BH (1992). Granulocyte-macrophage colony-stimulating factor (GM-CSF): a variety of possible applications in clinical medicine.. Infection.

[33] Tada R, Harada T, Nagi-Miura N, Adachi Y, Nakajima M (2007). NMR characterization of the structure of a beta-(1→3)-D-glucan isolate from cultured fruit bodies of *Sparassis crispa*.. Carbohydr Res.

[34] Tada R, Adachi Y, Ishibashi K, Ohno N (2009). An unambiguous structural elucidation of a 1,3-beta-D-glucan obtained from liquid-cultured *Grifola frondosa* by solution NMR experiments.. Carbohydr Res.

[35] Yuminamochi E, Koike T, Takeda K, Horiuchi I, Okumura K (2007). Interleukin-12- and interferon-gamma-mediated natural killer cell activation by *Agaricus blazei* Murill.. Immunology.

[36] Harada T, Miura NN, Adachi Y, Nakajima M, Yadomae T (2004). Granulocyte-macrophage colony-stimulating factor (GM-CSF) regulates cytokine induction by 1,3-beta-d-glucan SCG in DBA/2 mice in vitro.. J Interferon Cytokine Res.

[37] Harada T, Ohno N (2008). Contribution of dectin-1 and granulocyte macrophage-colony stimulating factor (GM-CSF) to immunomodulating actions of beta-glucan.. Int Immunopharmacol.

[38] Tada R, Yoshikawa M, Kuge T, Tanioka A, Ishibashi K (2011). Granulocyte macrophage colony-stimulating factor is required for cytokine induction by a highly 6-branched 1,3-beta-D-glucan from *Aureobasidium pullulans* in mouse-derived splenocytes.. Immunopharmacol Immunotoxicol.

[39] Akanuma AM, Motoi M, Yamagishi A, Ohno N (2006). Cloning and characterization of polyphenoloxidase DNA from *Agaricus brasiliensis* S. Wasser et al. (Agaricomycetideae).. Int J Med Mushr.

[40] Akanuma AM, Akanuma S, Motoi M, Yamagishi A, Ohno N (2011). Partial purification and characterization of polyphenoloxidase from culinary-medicinal Royal Sun mushroom (the Himematsutake), *Agaricus brasiliensis* S. Wasser et al. (Agaricomycetideae).. Int J Med Mushr.

[41] Dialynas DP, Wilde DB, Marrack P, Pierres A, Wall KA (1983). Characterization of the murine antigenic determinant, designated L3T4a, recognized by monoclonal antibody GK1.5: expression of L3T4a by functional T cell clones appears to correlate primarily with class II MHC antigen-reactivity.. Immunol Rev.

[42] Dialynas DP, Quan ZS, Wall KA, Pierres A, Quintans J (1983). Characterization of the murine T cell surface molecule, designated L3T4, identified by monoclonal antibody GK1.5: similarity of L3T4 to the human Leu-3/T4 molecule.. J Immunol.

[43] Wineman JP, Gilmore GL, Gritzmacher C, Torbett BE, Muller-Sieburg CE (1992). CD4 is expressed on murine pluripotent hematopoietic stem cells.. Blood.

[44] Vremec D, Pooley J, Hochrein H, Wu L, Shortman K (2000). CD4 and CD8 expression by dendritic cell subtypes in mouse thymus and spleen.. J Immunol.

[45] Graziani-Bowering G, Filion LG, Thibault P, Kozlowski M (2002). CD4 is active as a signaling molecule on the human monocytic cell line Thp-1.. Exp Cell Res.

[46] Kawai K, Tsuno NH, Kitayama J, Okaji Y, Yazawa K (2003). Epigallocatechin gallate, the main component of tea polyphenol, binds to CD4 and interferes with gp120 binding.. J Allergy Clin Immunol.

[47] Nakashima H, Murakami T, Yamamoto N, Naoe T, Kawazoe Y (1992). Lignified materials as medicinal resources. V. Anti-HIV (human immunodeficiency virus) activity of some synthetic lignins.. Chem Pharm Bull (Tokyo).

[48] Sakagami H, Oh-hara T, Kohda K, Kawazoe Y (1991). Lignified materials as a potential medicinal resource. IV. Dehydrogenation polymers of some phenylpropenoids and their capacity to stimulate polymorphonuclear cell iodination.. Chem Pharm Bull (Tokyo).

[49] Denizot F, Lang R (1986). Rapid colorimetric assay for cell growth and survival. Modifications to the tetrazolium dye procedure giving improved sensitivity and reliability.. J Immunol Methods.

